# Immunohistochemical Analysis of Cancer Stem Cell Marker Expression in Papillary Thyroid Cancer

**DOI:** 10.3389/fendo.2019.00523

**Published:** 2019-08-02

**Authors:** Hye Min Kim, Ja Seung Koo

**Affiliations:** Department of Pathology, Yonsei University College of Medicine, Seoul, South Korea

**Keywords:** cancer stem cell, papillary thyroid cancer, prognosis, CD15, thyroid gland

## Abstract

Cancer stem cell (CSC) markers have prognostic significance in various cancers, but their clinical significance in papillary thyroid carcinoma (PTC) has not been demonstrated. In this study, CSC markers expressed in PTC and their relationships with prognosis were evaluated. We constructed tissue microarrays for 386 PTC cases, divided it into 42 low risk cases and 344 intermediate risk cases according to the American Thyroid Association 2009 Risk Stratification System. Immunohistochemical staining of CSC markers (CD15, CD24, CD44, CD166, and ALDH1A1) was performed, and the proportion of stained cells and immunostaining intensity were evaluated to determine positive marker expression. The relationships between CSC marker expression and other clinicopathological parameters or survival were analyzed. CD15 expression was higher in PTC with intermediate risk than in PTC with low risk (29.4 vs. 11.9%, *p* = 0.017). According to a multivariate analysis, CD15, CD44, CD166, and ALDH1A1 positivity were independently associated with a shorter progression-free survival (PFS) (odds ratio [OR]: 1.929, 2.960, 7.485, and 3.736; *p* = 0.016, *p* = 0.026, *p* < 0.001, and *p* = 0.006, respectively). Higher N and cancer stage were the only other clinical factors associated with a shorter PFS (OR: 2.953 and 1.898, *p* = 0.011 and *p* = 0.034). Overexpression of CSC markers in PTC was associated with shorter PFS during follow-up. Immunohistochemical staining of CSC markers may provide useful information for predicting patient outcomes.

## Introduction

Thyroid cancer is the most common type of endocrine-related cancer, affecting 3.2 million people worldwide in 2015 (1). Among thyroid cancers, papillary thyroid cancer (PTC) accounts for 80–85% of cases. In general, the prognosis of PTC is favorable because of its low biological aggressiveness (2–4). However, in cases of disease recurrence or metastasis owing to a poor response or resistance to the standard treatment of thyroidectomy and radioactive iodine-131 therapy, patient death may occur and alternative treatment options should be sought. Therefore, numerous efforts have been made to identify markers of aggressive cancer behaviors. While the cause of aggressiveness in certain patients with PTC is unclear, several lines of evidence suggest an association with a rare subpopulation of tumor cells with stem cell-like features, also known as cancer stem cells (CSCs) (5, 6). CSCs have important roles in cancer development, growth, recurrence, and metastasis owing to their potential to self-renew and differentiate into various cells lineages. These characteristics may result in the formation of heterogenous tumor cell masses and the acquisition of resistance to chemotherapy and radiotherapy (7–10).

The role of CSCs in malignancy was first demonstrated in a leukemia model. While tumorigenesis is not provoked in immunodeficient non-obese diabetic/severe combined immunodeficiency mice upon transplantation of most tumor cells, the transplantation of certain tumor cells induces tumorigenesis (11). Subsequent studies have further evaluated the role of CSCs in various solid and hematological malignancies. For example, CSCs were found to be related to resistance to therapy and poor prognosis in patients with esophageal and breast cancer (12–15). In line with these findings, CSC marker expression is correlated with poor prognosis in colorectal carcinoma, suggesting that the expression of CSC markers is associated with patient prognosis in various cancers (16, 17).

Several surrogate marker of CSCs have been previously described. For example, CD24, CD44, and ALDHA1 have been suggested to be CSC markers in breast cancer (13), and CD166 has been implicated in colon cancer (17). In a thyroid cancer cell line, CD15, CD44, CD166, and ALDHA1 are markers of thyroid epithelial cell stemness (18). Moreover, Xu et al. have shown that SSEA-1 (CD15) immunoreactivity is associated with an aggressive subtype of thyroid carcinoma (19). However, the relationship between CSC marker expression and patient prognosis in PTC remains unclear. Therefore, the objective of this study was to investigate the expression of CSC markers in PTC and evaluate its clinical significance.

## Materials and Methods

### Cancer Stem Cell Marker Selection

To select candidate markers for CSCs, an *in silico* analysis and literature review were performed and the most extensively studied candidate markers were chosen (20, 21). CD44 expression was higher in thyroid cancer tissues than in normal thyroid tissues according to the Gene Expression across Normal and Tumor Tissue (GENT) web-accessible database (http://medical-genome.kribb.re.kr/GENT). The web-accessible database cBioPortal (http://www.cbioportal.org) was used to evaluate CSC marker abnormalities in thyroid cancer tissues (Supplementary Figure S1). As a result, CD15, CD24, CD44, CD166, and ALDH1A1 were selected as markers of CSCs in this study.

### Patient Selection

A total of 386 patients pathologically diagnosed with PTC at Severance Hospital who underwent the surgical removal of cancer and for whom paraffin blocks were available were recruited. Patients were divided into two groups, low risk (*n* = 42) and intermediate risk (*n* = 344), according to the American Thyroid Association 2009 Risk Stratification System (22). All cases were retrospectively reviewed by a thyroid pathologist (JSK), and histological evaluation was performed after hematoxylin and eosin staining. Clinicopathological data were obtained from medical records and included age at diagnosis, sex, disease recurrence/metastasis, and all-cause mortality. The T, N, and cancer stage (23), margin (expanding or infiltrative), extent (confined to the thyroid parenchyma or with extrathyroidal spread), and presence of *BRAF* V600E mutations were also noted after reviewing the slides and surgical pathology reports. This study was approved by the Institutional Review Board of Severance Hospital and conducted in accordance with the principles set forth in the Declaration of Helsinki. The requirement to obtain informed consent was waived from the Institutional Review Board of Severance Hospital because this was a retrospective study.

### Tissue Microarray

Representative areas were selected on hematoxylin and eosin-stained slides, and a corresponding spot was marked on the surface of the matching paraffin block. Tissue microarrays (TMAs) were constructed from representative tissue columns for the 386 PTC cases. Three-millimeter tissue cores were extracted from the selected areas using a manual tissue arrayer and placed into a 6 × 5 recipient block. Two tissue cores were extracted from each sample to minimize extraction bias. Each tissue core was assigned a unique TMA location number, which was linked to a database containing other clinicopathological data.

### Immunohistochemistry

Antibodies used for immunohistochemistry are listed in Supplementary Table S1. All immunohistochemical analyses were performed with formalin-fixed, paraffin-embedded tissue sections using an automatic immunohistochemistry staining device (Benchmark XT; Ventana Medical System, Tucson, AZ, USA). Briefly, 5-μm-thick formaldehyde-fixed, paraffin-embedded tissue sections were transferred to adhesive slides and dried at 62°C for 30 min. Standard heat epitope retrieval was performed for 30 min in ethylene diamine tetraacetic acid, pH 8.0, in an autostainer. The samples were then incubated with primary antibodies. Afterwards, the sections were incubated with biotinylated anti-mouse immunoglobulins, peroxidase-labeled streptavidin (LSAB Kit, DakoCytomation, Agilent, Santa Clara, CA, USA), and 3,3′-diaminobenzidine. Negative control samples were processed without the primary antibody. Positive control tissues were used per the manufacturer's recommendation. Slides were counterstained with Harris hematoxylin. Optimal primary antibody incubation times and concentrations were determined by serial dilutions using a tissue block fixed and embedded exactly as performed for the samples.

### Interpretation of Immunohistochemical Staining

Immunohistochemical markers were assessed by light microscopy. The stained slides were semi-quantitatively evaluated as described previously (24). Staining was evaluated by calculating the proportion of stained cells and immunostaining intensity. The immunostaining intensity was defined as follows: 0, negative; 1, weak; 2, moderate; and 3, strong. The scores for the proportion of stained cells and immunostaining intensity were multiplied, and staining was defined as positive when the final score was >10. *BRAF* V600E mutation status was evaluated using immunohistochemical staining, and was considered positive when >20% of tumor cells were positive (25).

### Statistical Analysis

All data are presented as frequencies and percentages. Data were compared using the Chi-squared test or Fisher's exact test, as appropriate. Associations between the proportion of CSC marker-stained cells and *BRAF* V600E mutation status were analyzed using Spearman's rho. Univariate and multivariate Cox proportional hazard analyses were used to evaluate prognostic factors for progression-free survival (PFS) and overall survival (OS). For all statistical analyses, a two-tailed *p*-value of <0.05 was considered statistically significant. Data analyses were performed using IBM SPSS Statistics for Windows, Version 21.0 (Released 2012; IBM Corp., Armonk, NY, USA).

## Results

### Baseline Characteristics of Patients

The clinicopathological features of patients included in this study are presented in Table 1. Among the patients, 305 (79.0%) were female, and the mean age was 47.0 years (range: 20–82 years). The N and cancer stages were higher in patients in the intermediate risk group than in those in the low risk group. Moreover, infiltrative margins, extrathyroidal extension, and *BRAF* V600E mutation were more frequently observed in PTC with intermediate risk than in the low risk group. No significant differences were found with respect to age, gender, T stage, recurrence/metastasis, and mortality between the two groups.

**Table 1:** Clinicopathological features of patients with papillary thyroid carcinoma according to the American Thyroid Association 2009 Risk Stratification System.

	**Total *N* = 386 (%)**	**ATA low risk group *n* = 42 (%)**	**ATA intermediate risk group *n* = 344 (%)**	***p-*value**
Age (years)				0.370
<45	168 (43.5)	21 (50.0)	147 (42.7)	
≥45	218 (56.5)	21 (50.0)	197 (57.3)	
Sex				0.634
Male	81 (21.0)	10 (23.8)	71 (20.6)	
Female	305 (79.0)	32 (76.2)	273 (79.4)	
T stage				0.260
T1	279 (72.3)	33 (78.6)	246 (71.5)	
T2	100 (25.9)	9 (21.4)	91 (26.5)	
T3	7 (1.8)	0 (0.0)	7 (2.0)	
*N* stage				**<0.001**
N0	144 (37.3)	28 (66.7)	116 (33.7)	
N1	242 (62.7)	14 (33.3)	228 (66.3)	
Cancer stage				**0.006**
I	268 (69.4)	37 (88.1)	231 (67.2)	
II	118 (30.6)	5 (11.9)	113 (32.8)	
Expanding margin				**<0.001**
No	328 (85.0)	19 (45.2)	309 (89.8)	
Yes	58 (15.0)	23 (54.8)	35 (10.2)	
Tumor extension				**<0.001**
Intrathyroidal	110 (28.5)	42 (100.0)	68 (19.8)	
Extrathyroidal	276 (71.5)	0 (0.0)	276 (80.2)	
*BRAF* V600E mutation (*n* = 320)^*^				**<0.001**
No	95 (29.7)	30 (100.0)	65 (22.4)	
Yes	225 (70.3)	0 (0.0)	225 (77.6)	
Recurrence/metastasis				0.792
No	327 (84.7)	35 (83.3)	292 (84.9)	
Yes	59 (15.3)	7 (16.7)	52 (15.1)	
Mortality				0.246
No	367 (95.1)	42 (100.0)	325 (94.5)	
Yes	19 (4.9)	0 (0.0)	19 (5.5)	

* *Cases that were not tested for the BRAF V600E mutation were excluded. ATA, American thyroid association. Bold values indicate statistically significant (p <0.05)*.

### Expression of CSC Markers in the Low and Intermediate Risk Groups

**Figure 1:**
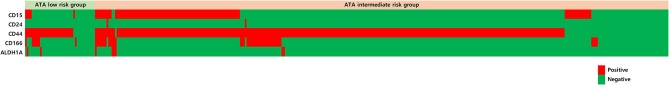
Heat map of cancer stem cell markers expressed in papillary thyroid carcinoma with ATA low risk and ATA intermediate risk.

**Figure 2:**
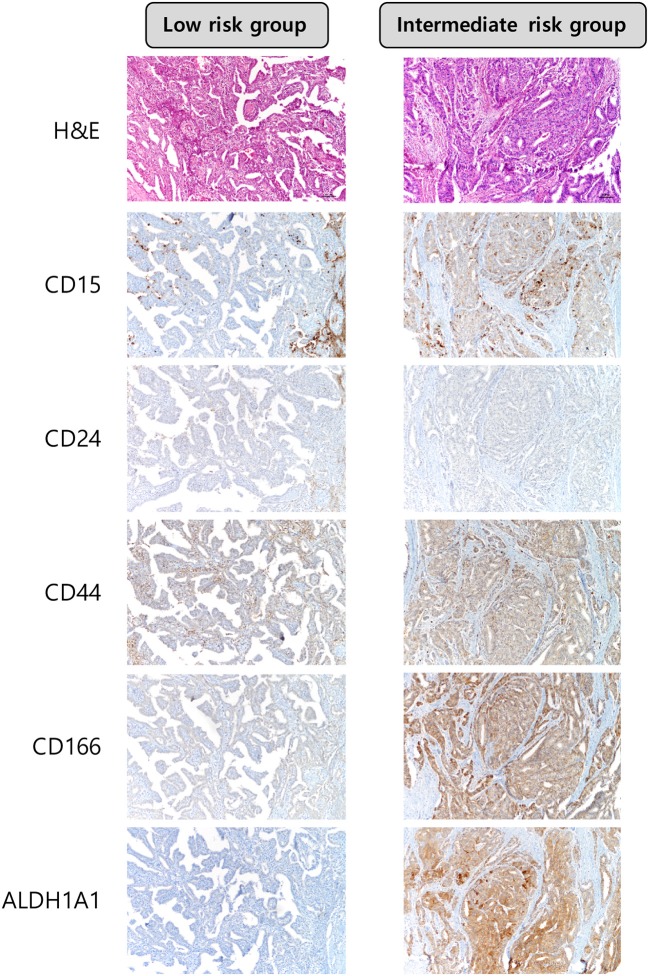
Expression of cancer stem cell markers in papillary thyroid carcinoma with ATA low risk and ATA intermediate risk. The expression of CD15 was higher in PTCs with ATA intermediate risk × 100.

Among the CSC markers, CD44 positivity was most frequent, followed by CD166 positivity and CD15 positivity. The expression of CD15 was significantly higher in the intermediate risk group than in the low risk group (29.4 vs. 11.9%, *p* = 0.017; Table 2). CD24 positivity was found in only 2 (0.5%) cases, and there was no statistical difference in the expression of CD24 between the two groups. A heat map of CSC marker expression and representative images of immunohistochemical staining of CSC markers in PTC with low risk and intermediate risk are shown in Figures 1, 2.

**Table 2:** Expression of cancer stem cell markers according to the American Thyroid Association 2009 Risk Stratification System.

	**Total *N* = 386 (%)**	**ATA low risk group *n* = 42 (%)**	**ATA intermediate risk group *n* = 344 (%)**	***p*-value**
CD15				**0.017**
Negative	280 (72.5)	37 (88.1)	243 (70.6)	
Positive	106 (27.5)	5 (11.9)	101 (29.4)	
CD24				0.999
Negative	384 (99.5)	42 (100.0)	342 (94.4)	
Positive	2 (0.5)	0 (0.0)	2 (0.6)	
CD44				0.052
Negative	76 (19.7)	13 (31.0)	63 (18.3)	
Positive	310 (80.3)	29 (69.0)	281 (81.7)	
CD166				0.347
Negative	339 (87.8)	35 (83.3)	304 (88.4)	
Positive	47 (12.2)	7 (16.7)	40 (11.6)	
ALDH1A1				0.212
Negative	378 (97.9)	40 (95.2)	338 (98.3)	
Positive	8 (2.1)	2 (4.8)	6 (1.7)	

*ATA, American thyroid association. Bold value indicates statistically significant (p <0.05)*.

### Correlations Between CSC Marker Expression and Clinicopathological Factors in PTC

**Figure 3:**
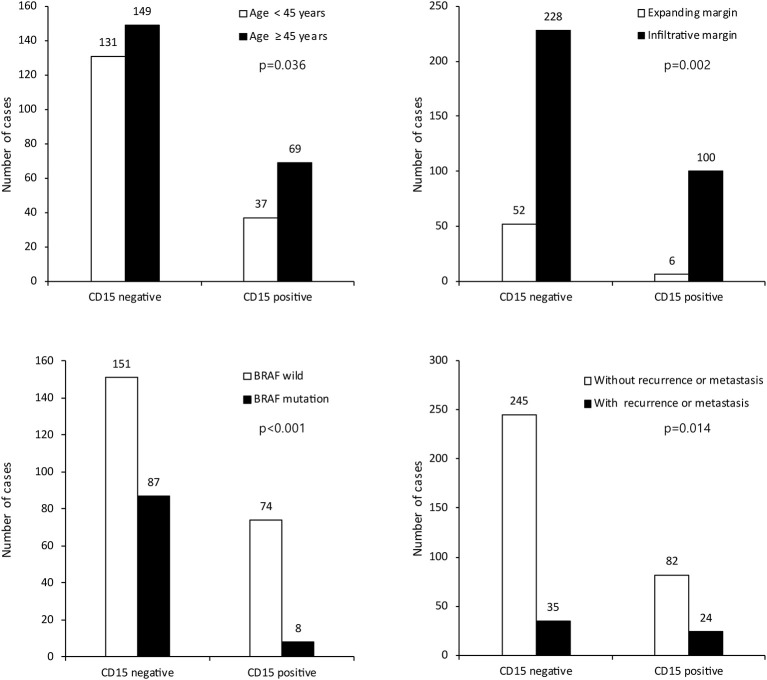
Expression of cancer stem cell marker CD15 and its association with clinicopathologic factors.

**Figure 4:**
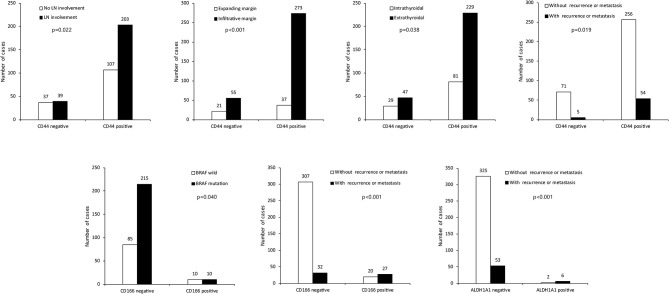
Expression of cancer stem cell markers CD44, CD166, ALDH1A1, and clinicopathological factors. LN, lymph node.

We investigated the correlations between the expression of CSC markers and clinicopathological factors in PTC. CD15 positivity was associated with older age (≥45 years; *p* = 0.036), infiltrative margins (*p* = 0.002), *BRAF* V600E mutation (*p* < 0.001), and recurrence/metastasis (*p* = 0.014; Figure 3). Similarly, CD44 positivity was associated with a higher N stage (*p* = 0.022), infiltrative margins (*p* < 0.001), extrathyroidal involvement (*p* = 0.038), and recurrence/metastasis (*p* = 0.019). CD166 positivity was associated with *BRAF* V600E mutation and recurrence/metastasis (*p* = 0.040 and *p* < 0.001, respectively), and ALDH1A1 positivity was associated with recurrence/metastasis (*p* < 0.001; Figure 4).

### Correlations Between CSC Markers and *BRAF* V600E Mutation Status

The *BRAF* V600E mutation status was evaluated by immunohistochemistry in 320 cases of PTC. In total, 225 of the cases (70.3%) exhibited the *BRAF* V600E mutation. The proportions of CD15- and CD24-stained cells were correlated with the *BRAF* V600E mutation status (*r* = 0.393, *p* < 0.001 and *r* = 0.124, *p* = 0.026; Table 3). An association between CD15-stained cells and *BRAF* V600E mutation status was also observed in PTC cases with lymph node metastasis (*r* = 0.367, *p* < 0.001). However, no significant correlations were observed between the frequencies of the remaining CSC markers and the *BRAF* V600E mutation status.

**Table 3:** Correlations between cancer stem cell marker expression and *BRAF* V600E mutation status.

**Parameters**	**Total (*****n*** **= 320)**		**PTC with lymph node metastasis (*****n*** **= 188)**	
	**Correlation coefficient**	***p*-value**	**Correlation coefficient**	***p*-value**
CD15	0.393	**<0.001**	0.367	**<0.001**
CD24	0.124	**0.026**	0.111	0.130
CD44	−0.050	0.369	−0.050	0.497
CD166	−0.045	0.427	−0.095	0.194
ALDH1A1	−0.024	0.668	−0.052	0.481

*PTC, papillary thyroid carcinoma. Bold values indicate statistically significant (p <0.05)*.

### CSC Marker Expression Is Associated With Disease Recurrence or Metastasis in PTC

We investigated the impact of clinical parameters and CSC marker expression on the clinical outcomes of patients with PTC using a Cox proportional hazard analysis. The mean follow-up period was 79.1 months, with 59 patients (15.3%) suffering recurrence or metastasis and 19 patients (4.9%) dying during the follow-up period. According to a univariate analysis, higher N and cancer stages, extrathyroidal involvement, and CD15, CD44, CD166, and ALDH1A1 positivity were related to a shorter PFS. A multivariate analysis revealed that a higher N stage [odds ratio (OR): 2.953, 95% confidence interval (CI): 1.286–6.783, *p* = 0.011], higher cancer stage (OR: 1.898, 95% CI: 1.050–3.430, *p* = 0.034), CD15 positivity (OR: 1.929, 95% CI: 1.132–3.288, *p* = 0.016), CD44 positivity (OR: 2.960, 95% CI: 1.137–7.704, *p* = 0.026), CD166 positivity (OR: 7.485, 95% CI: 4.333–12.930, *p* < 0.001), and ALDH1A1 positivity (OR: 3.736, 95% CI: 1.467–9.515, *p* = 0.006) were independently associated with a shorter PFS. However, only a higher cancer stage (OR: 6.839, 95% CI: 2.462–19.000, *p* < 0.010) was associated with a shorter OS according to univariate and multivariate analyses (Table 4).

**Table 4:** Cox proportional hazard analysis of progression-free survival and overall survival in papillary thyroid carcinoma.

	**Progression free survival**						**Overall survival**					
	**Univariate analysis**			**Multivariate analysis**			**Univariate analysis**			**Multivariate analysis**		
	**Odds ratio**	**95% CI**	***p-*value**	**Odds ratio**	**95% CI**	***p*-value**	**Odds ratio**	**95% CI**	***p-*value**	**Odds ratio**	**95% CI**	***p-*value**
Age ≥ 45 years	1.358	0.801–2.302	0.256				14.244	1.901–106.704	**0.010**			
Male sex	1.690	0.971–2.942	0.064				0.678	0.197–2.326	0.536			
Diameter ≥ 2.0 cm	1.567	0.925–2.656	0.095				2.299	0.934–5.659	0.070			
T stage	1.317	0.828–2.096	0.245				1.752	0.824–3.723	0.145			
N stage	4.253	2.018–8.967	**<0.001**	2.953	1.286–6.783	**0.011**	1.786	0.643–4.966	0.266			
Cancer stage	2.487	1.492–4.146	**<0.001**	1.898	1.050–3.430	**0.034**	6.839	2.462–19.000	**<0.001**	6.839	2.462–19.000	**<0.001**
Infiltrative margin	1.678	0.721–3.904	0.230				3.416	0.456–25.607	0.232			
Extrathyroidal involvement	2.025	1.026–3.998	**0.042**				0.860	0.327–2.262	0.759			
CD15 positivity	1.943	1.155–3.267	**0.012**	1.929	1.132–3.288	**0.016**	1.616	0.636–4.109	0.313			
CD24 positivity	n/a	n/a	n/a				n/a	n/a	n/a			
CD44 positivity	2.917	1.167–7.294	**0.022**	2.960	1.137–7.704	**0.026**	0.999	0.331–3.016	0.999			
CD166 positivity	8.056	4.810–13.494	**<0.0001**	7.485	4.333–12.930	**<0.001**	0.428	0.057–3.212	0.410			
ALDH1A1 positivity	7.236	3.105–16.861	**<0.001**	3.736	1.467–9.515	**0.006**	n/a	n/a	n/a			

*CI, Confidence interval; n/a, not applicable. Bold values indicate statistically significant (p <0.05)*.

## Discussion

In this study, we evaluated the expression of CSC markers in PTC and their correlations with clinicopathological parameters and prognosis. We found that in PTC with intermediate risk, the expression of the CSC marker CD15 was significantly elevated. Furthermore, the CSC markers CD15, CD44, CD166, and ALDH1A1 were associated with an aggressive phenotype and were each independently associated with a shorter PFS. The findings of our study are consistent with those of previous studies demonstrating that CSC marker expression is associated with aggressive biological features in various cancers (26–28). Collectively, our findings suggest that CSC markers are potential biological markers for PTC.

In a previous study, CD15 positivity was associated with poor survival in anaplastic thyroid cancer (19). Furthermore, Han et al. have demonstrated that CD24 and CD44 are associated with aggressive clinicopathological features in PTC (29). Together, these findings suggest that the expression of CSC markers may be associated with prognosis. In this study, among the five CSC markers analyzed, we found that CD15, CD44, CD166, and ALDH1A1 expression were independently associated with a shorter PFS. These results provide the first evidence that the expression of CSC markers is associated with patient prognosis in PTC. However, in a Cox proportional hazard analysis, only a higher cancer stage, but not CSC marker expression, was related to patient OS. This may be explained by the nature of PTC, which is relatively indolent compared to other malignancies (3, 4).

Several cellular pathways may mediate the link between CSCs and the pathogenesis of PTC, namely the Notch, Hedgehog (Hh), and Wnt/β-catenin pathways. The Notch pathway is known to facilitate the self-renewal of CSCs in various cancers and is involved in the interaction between the tumor stroma and the endothelium of the CSC microenvironment (30, 31). The Hh pathway is reportedly related to the maintenance of CSCs (32), and the activation of this pathway in cancers is associated with the development of resistance to chemotherapy or radiotherapy (33). The Wnt/β-catenin pathway has also been suggested to play an important role in CSC maintenance (34). Interestingly, a previous study has demonstrated that in PTC, the activation of molecules related to the β-catenin pathway in isolated CSCs promoted cancer migration and metastasis (34). Furthermore, loss-of-function mutations in adenomatosus polyposis coli is associated with CSC activation via β-catenin activation and enhancement of KRAS mutation in colorectal cancer tumorigenesis (35). Because RAS mutation has also been identified in PTC as well as BRAF mutation, these findings altogether suggest an association between CSC and PTC (36).

In a correlation analysis, the expression of the CSC markers CD15 and CD24 was significantly correlated with the *BRAF* V600E mutation status. In PTC, the *BRAF* V600E mutation is reportedly associated with extrathyroidal extension, multifocality, advanced cancer stage, lymph node metastasis, and recurrence (37). A possible explanation for the association between the *BRAF* V600E mutation status and the expression of CSC markers may involve c-MYC and HIF-1α, which are downstream molecules of the mitogen-activated protein kinase pathway. In human ovarian cancer cells, HIF-1α promotes CSC-like properties by upregulating SIRT1 expression (38). In addition, c-MYC overexpression leads to a significant increase in CSCs in breast cancer cell lines (39). Interestingly, Han et al. (29) recently showed that the *BRAF* V600E mutation is associated with CD44 expression in PTC with lymph node metastasis. However, in our study, a significant relationship between CD44 expression and the *BRAF* V600E mutation status was not observed. This difference among studies can likely be attributed to the difference in antibodies used for immunohistochemistry and differences in patient clinical characteristics; further studies are required to evaluate the association between the *BRAF* V600E mutation and the expression of CSC markers.

Our study has several important clinical implications. First, our findings suggest that CSC markers may have prognostic value in PTC. Lymph node involvement, infiltrative margins, and lymphovascular invasion are pathological factors traditionally associated with disease recurrence or metastasis in PTC (40). However, other biological markers reflecting disease recurrence or metastasis are largely unclear. Although a majority of PTCs are indolent in nature, the prognosis for PTC with recurrence or metastasis could be unfavorable (41, 42). Accordingly, it is necessary to identify markers for the identification of patients with high risk of disease recurrence or metastasis. Currently, the American Thyroid Association 2009 Risk Stratification System is the most widely accepted recommendation regarding the initial management of thyroid cancer, which includes cancer screening, staging and risk assessment, and treatment (22). Of note, in the present study, the expression of CD15 was significantly higher in the ATA intermediate risk group than in the low risk group. Moreover, CD15, CD44, CD166, and ALDH1A1 positivity were each independently associated with a shorter PFS, independently of higher N and cancer stage as well as other clinicopathological factors. These findings suggest that CSC markers are ancillary biological markers for evaluating patient prognosis in PTC. Second, CSCs may be potential therapeutic targets for the treatment of PTC. The role of CSCs in PTC tumorigenesis remains uncertain. However, owing to their important functions in tumor initiation and the development of treatment resistance, targeting CSCs may be useful for preventing cancer recurrence or metastasis (43, 44). Currently, the first-line therapy for PTC mainly consists of thyroidectomy and radioactive iodine-131 therapy, which targets mature tumor cells; however, in iodine treatment-refractory cases with disease recurrence or metastasis, chemotherapy using tyrosine kinase inhibitors is recommended (45). Nevertheless, considering the aggressive nature and the difficulty in controlling PTC with recurrence or metastasis (41), there is a need for additional therapeutic options. Targeting CSCs in PTC is a promising strategy for preventing tumor growth, invasion, and metastasis; however, this should be confirmed in clinical trials.

There are several limitations of this study. First, we used immunohistochemistry to evaluate the expression of CSC markers; the quantification of results is therefore difficult and there is the potential for inter-observer bias. Second, as a TMA was used rather than whole sections for histological examination, samples may have been influenced by extraction bias during TMA construction. Third, we were not able to evaluate molecular changes or the mechanisms underlying CSC marker expression in thyroid cancer tissues, as we focused on expression at the protein level. Fourth, the study was retrospective and selection bias is possible. Further experiments are therefore necessary to elucidate the expression of CSC markers at the gene level and their contributions to the pathogenesis of the disease.

In conclusion, we found that the expression of the CSC marker CD15 was higher in the ATA intermediate risk group than in the low risk group and that the expression of CSC markers is associated with more aggressive tumor characteristics and poor prognosis, thus providing a rationale to evaluate CSC markers in PTC.

## Ethics Statement

This study was approved by the Institutional Review Board of Severance Hospital and conducted in accordance with the principles set forth in the Declaration of Helsinki. The requirement to obtain informed consent was waived from Institutional Review Board of Severance Hospital because this study was performed retrospectively.

## Author Contributions

JK and HK analyzed the data, conceived of and designed the experiments. HK performed the experiments. JK contributed reagents, materials, analysis tools. HK wrote the paper. Every author has taken care to ensure the integrity of this work, and the final manuscript has been seen and approved by all authors before submission.

### Conflict of Interest Statement

The authors declare that the research was conducted in the absence of any commercial or financial relationships that could be construed as a potential conflict of interest.
