# Supplementary material for: Immunohistochemical Analysis of Cancer Stem Cell Marker Expression in Papillary Thyroid Cancer
Source: Front Endocrinol (Lausanne). 2019 Aug 2;10:523. doi: 10.3389/fendo.2019.00523 (PMC6688385; doi:10.3389/fendo.2019.00523)
Supplement: Supplementary file 1 [file Data_Sheet_1.docx]

**Supplementary Figure S1.** CD44 expression in human thyroid cancers. (A) CD44 expression in thyroid cancer (C) and normal thyroid (N) tissues according to public data retrieved from GENT (http://medical-genome.kribb.re.kr/GENT). The GSE2109 data set was used for analysis. Each circle represents an individual tissue sample. (B) CD44 abnormalities in thyroid cancers according to cBioPortal data (http://www.cBioPortal.org).


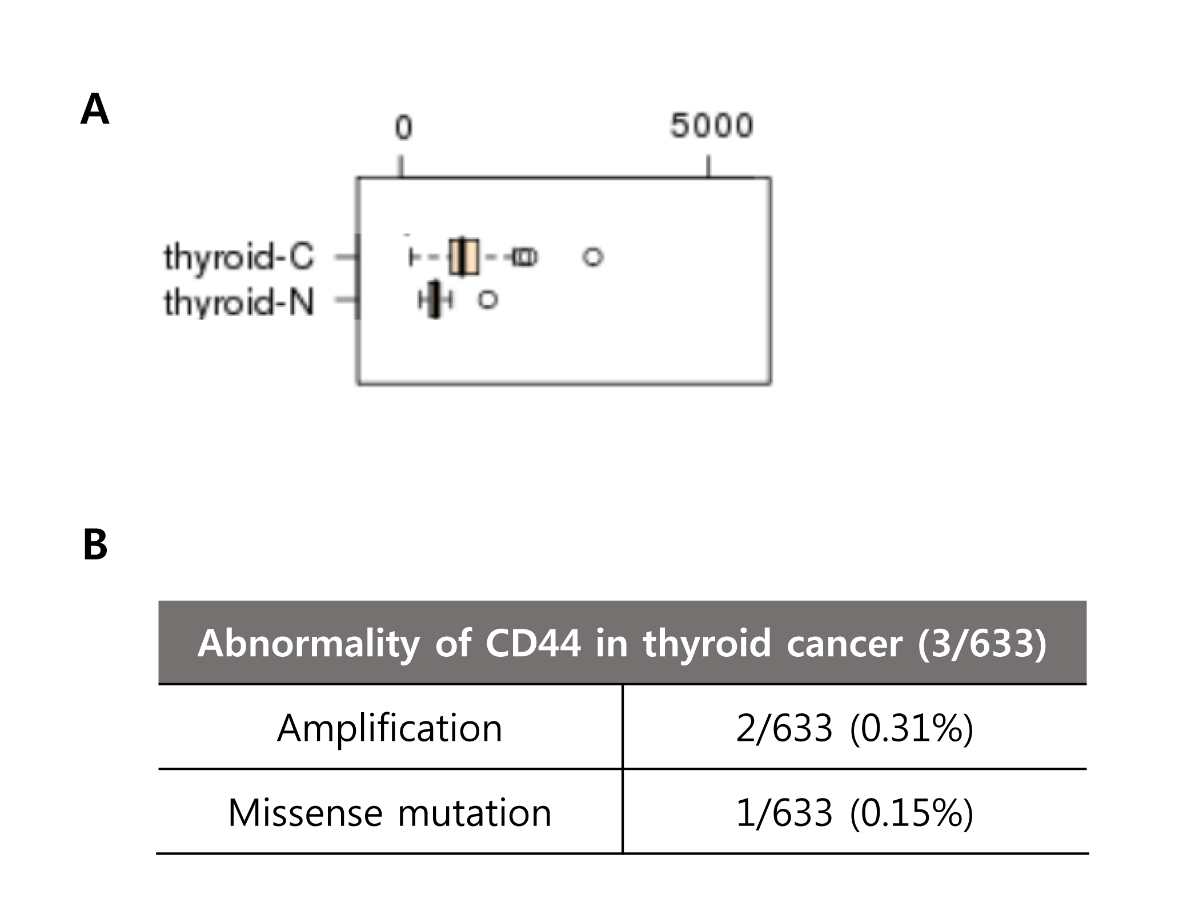


| **Supplementary table S1.** Source, clone, and dilution of antibodies used in this study. | | | |
| --- | --- | --- | --- |
| **Antibody** | **Clone** | **Dilution** | **Company** |
| CD15 | MC-480 | 1:100 | Abcam, Cambridge, UK |
| CD24 | SN3b | 1:50 | Thermo Fisher, Rockford, USA |
| CD44 | EPR1013Y | 1:100 | Abcam, Cambridge, UK |
| CD166 | EPR2759(2) | 1:100 | Abcam, Cambridge, UK |
| ALDH1A1 | EP1933Y | 1:100 | Abcam, Cambridge, UK |
| BRAF V600E | VE1 | 1:50 | Ventana, Tucson, AZ, USA |
